# What is the optimal prolactin cutoff for predicting the presence of a pituitary adenoma in patients with polycystic ovary syndrome?

**DOI:** 10.7150/ijms.80891

**Published:** 2023-02-13

**Authors:** Sang Il Kim, Joo Hee Yoon, Dong Choon Park, Seung Ho Yang, Young Il Kim

**Affiliations:** 1 Department of Obstetrics and Gynecology, St. Vincent's Hospital, College of Medicine, The Catholic University of Korea, Seoul, Republic of Korea.; 2 Department of Neurosurgery, St. Vincent's Hospital, College of Medicine, The Catholic University of Korea, Seoul, Republic of Korea.

**Keywords:** Polycystic ovary syndrome. Pituitary neoplasms, Prolactinoma, Hyperprolactinemia, Infertility.

## Abstract

**Objective:** Hyperprolactinemia (HPRL) and polycystic ovary syndrome (PCOS) are common causes of infertility in women of reproductive age. A pituitary adenoma (PA) is the most common type of brain tumor that causes HPRL. In the neurosurgical field, the co-existence of PA and PCOS is not common. However, neurosurgeons often treat patients who are referred from gynecology. Because most of these patients are young and reproductive-aged, it is difficult for a neurosurgeon to come up with a treatment plan alone. In this study, we investigated the prevalence of PAs in PCOS patients, the cutoff prolactin (PRL) level to detect PAs, and the treatment strategy, then assessed the relationship between these diseases via a literature review.

**Methods:** Medical records from November 2009 to March 2020 were reviewed at our institute. A total of 657 PCOS patients were enrolled. Initial prolactin levels were investigated and hyperprolactinemic patients were selected. As a result of sella magnetic resonance imaging (MRI), patients were divided into 2 groups of those with hyperprolactinemia but without PAs (group A) and those with both hyperprolactinemia and PAs (group B), respectively. We then compared and analyzed each group to find the characteristics and statistical differences. Receiver operating characteristic (ROC) curve analysis was performed to determine a cutoff value of the serum PRL level that could detect PAs in hyperprolactinemic PCOS patients.

**Results:** Of 657 patients diagnosed with PCOS, 76 patients had hyperprolactinemia (76/657, 11.6%). Sella MRI was performed in 56 patients, excluding 20 patients for various reasons. Patients in groups A and B numbered 43 and 13, respectively, and the mean serum prolactin level significantly differed between the groups (39.89 ± 41.64 vs. 108.59 ± 60.70 ng/mL, *P* < 0.001). Based on the ROC curve analysis of the prolactin threshold level for predicting PAs in PCOS patients, the area under the ROC curve was 0.853 (95% confidence interval, 0.733-0.934; *P* < 0.001), and the sensitivity and specificity were 76.9% and 86.1%, respectively. Ultimately, the cutoff value for prolactin level was 52.9 ng/mL.

**Conclusion:** PCOS and hyperprolactinemia are common causes of infertility in reproductive-age women. PCOS patients with a PRL level of ≥ 52.9 ng/mL may need to undergo sella MRI for detecting PAs. To help ensure a favorable clinical course for these patients, systematic diagnosis, treatment, and follow-up plan should be established. Therefore, a multidisciplinary approach involving both neurosurgery and gynecology is essential.

## Introduction

PCOS is one of the most common endocrine disorders in women of reproductive ages, with a prevalence of 6%-10% 1, 2. The 2003 Rotterdam Consensus Workshop revised the diagnostic criteria of PCOS and concluded that PCOS is a syndrome of ovary dysfunction with the cardinal features of hyperandrogenism (HA) and polycystic ovary morphology 3. In this new schema, PCOS remains a diagnosis of exclusion with the need to first rule out other disorders that mimic the PCOS phenotype, one of which is hyperprolactinemia (HPRL) 4. Many studies have documented modest HPRL in PCOS patients, with a prevalence of 11%-17% 5-8. Generally, this condition is regarded as functional in PCOS.

There are various causes of HPRL in reproductive-age women. Prolactinoma is the most common cause of HPRL other than physiological conditions such as pregnancy and lactation, which account for 40% of all pituitary tumors 9. A mild-to-moderate elevation of the prolactin level may occur in non-functioning pituitary adenoma (PA) (NFPA) or in prolactinoma due to the stalk-section effect 10.

The association between PCOS and PA is rare, and evidence to suggest the details of the relationship is lacking 11-13. At this time, there is no clear cutoff level of prolactin to differentiate between HPRL caused by PA and functional HPRL in PCOS without PA.

In this study, we investigated the prevalence of HPRL and PA in PCOS patients, the cutoff PRL level for detecting PA, and treatment strategies. We also attempted to investigate the association between PCOS and PA through a literature review.

## Materials and Methods

### Diagnosis of PCOS and patient enrollment

Medical records from November 2009 to March 2020 were reviewed at our institute, and a total of 657 patients diagnosed with PCOS with serum PRL measurements were enrolled. The diagnosis of PCOS was performed in the presence of ≥2 of the following criteria by a gynecologic clinician based on the Rotterdam criteria 3: ovulation dysfunction or oligo/amenorrhea, clinical HA and/or biochemical HA, and polycystic ovary morphology on ultrasonography.

### Prolactin assay information

The prolactin level was measured using the Dxl 800 Access immunoassay system analyzer with Access prolactin reagent (Beckman Coulter, Brea, CA, USA). This reagent is specified to avoid measuring macroprolactin so that an increase in prolactin level due to macroprolactinemia can be ruled out.

### Patient grouping

If HPRL (serum prolactin level ≥ 25.0 ng/mL) 9 was detected during the initial laboratory exam at the gynecologic department, the patient was referred to the neurosurgery department after excluding pregnancy, hypothyroidism, and long-term use of drugs that can cause HPRL. Sella magnetic resonance imaging (MRI) was performed in all cases except when the patient refused the MRI examination or dropped out during follow-up. Patients were then divided in 2 groups of those with HPRL but without PAs (group A) and those with both HPRL and PAs (group B).

### Collected data

 The details of patient age, body mass index (BMI), initial PRL level, clinical HA (%), menstrual irregularities (%), and PCO morphology (%) were collected in both groups. Information on tumor size, prolactin normalization (%), recovery of the menstruation (%), treatment modality, and follow-up period was additionally collected in group B.

### Statistical analysis

Fisher's exact test and the chi-squared test were used to compare categorical variables. Continuous variables were compared using Student's *t* test or the Mann-Whitney *U* test. Receiver operating characteristic (ROC) curve analysis was performed to determine a cutoff value for the serum PRL level that could detect PAs in hyperprolactinemic PCOS patients. All statistical analyses were performed using the MedCalc® Statistical Software version 20.112 (MedCalc Software Ltd., Ostend, Belgium). Statistical significance was set at <0.05.

## Results

**Figure 1:**
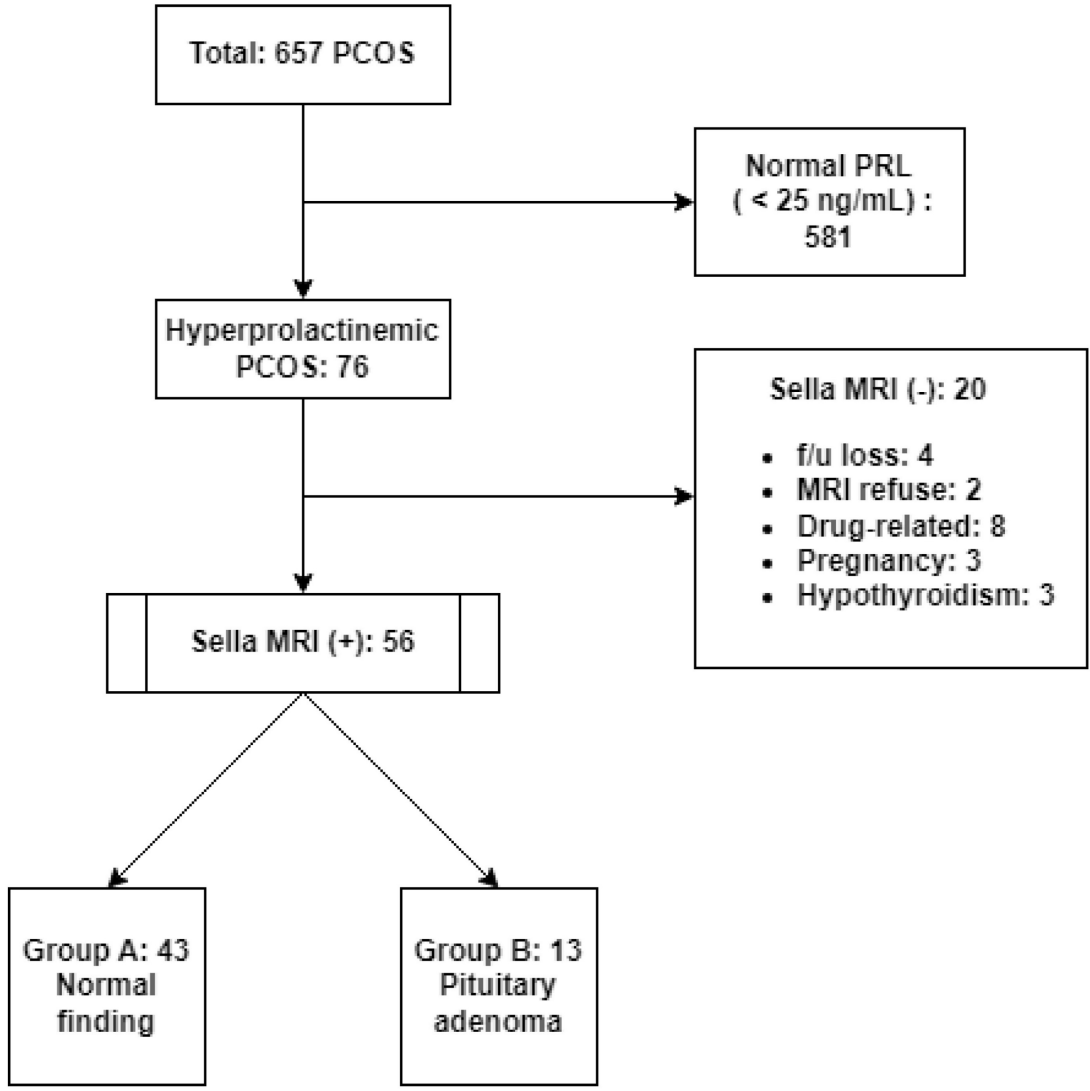
** Patient grouping flow chart.** PCOS, polycystic ovary syndrome; PRL, prolactin; MRI, magnetic resonance imaging; f/u: follow-up.

Of the total 657 patients, 581 had a normal PRL level (<25 ng/mL) and 76 patients were determined to have HPRL (76/657, 11.6%). Sella MRI was not performed in 20 of these HPRL patients for various reasons, and sella MRI was finally performed in 56 HPRL patients. A total of 43 patients with no confirmed PAs on MRI scans were classified as group A, while 13 patients with confirmed PAs were classified as group B (Fig. 1).

### Comparison between groups for clinicopathological characteristics

The clinicopathological characteristics of 56 patients are summarized in Table 1. The mean serum PRL level significantly differed between groups (39.89 ± 41.64 vs. 108.59 ± 60.70 ng/mL, *P* < 0.001). No significant differences were found between groups with regard to age or BMI. Additionally, no differences were observed in the frequency of clinical HA, menstrual irregularities, or polycystic ovary morphology between the groups.

### Analysis of group B patients (with hyperprolactinemic PCOS and PAs)

We conducted an additional data investigation of group B patients. The overall follow-up period was 3-84 (median, 22) months. The numbers of microadenoma (tumor size < 10 mm) and macroadenoma (tumor size > 10 mm) diagnosed were 9 and 4, respectively. Normalization of the PRL level and recovery of the menstruation were achieved in 12 patients (12/13, 92.3%) and 10 patients (10/13, 76.9%), respectively. One patient whose PRL level did not normalize had a microadenoma (size, 7 mm) and an initial PRL level of 103.2 ng/mL. The follow-up period was 84 months, during which time this patient was treated using only clomiphene by the gynecologist, and they experienced 2 pregnancies despite their prolactin level remaining at 79.3 ng/mL. Considering menstrual recovery, 1 patient dropped out of the study after being diagnosed with a PA and could not be assessed further. The prolactin level normalized in the other 2 patients, but their menstrual cycles did not recover. Dopamine receptor agonists (bromocriptine or cabergoline) were administered to 9 patients, excluding 3 who could not continue taking the drug due to side effects. Surgical intervention was performed in only 1 patient with a macroadenoma (size, 29 × 30 × 19 mm^3^), a PRL level of 26.7 ng/mL, and a visual field defect. Thereafter, her prolactin level was normalized, and menstrual recovery was achieved during the follow-up period. These patients received cooperative care involving the neurosurgeon, gynecologist, and endocrinologist.

### Cutoff value of prolactin for detecting PAs

**Figure 2:**
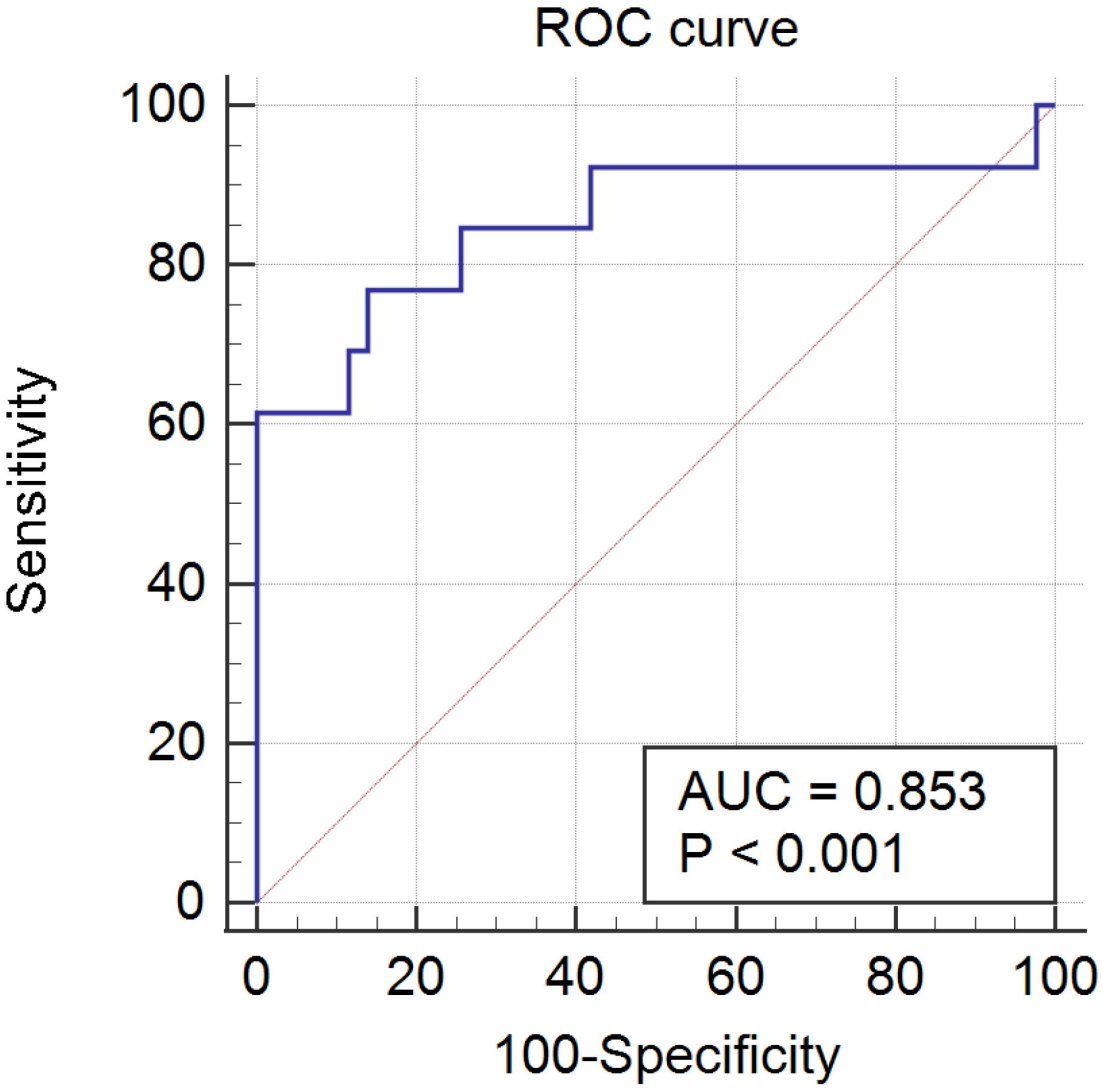
**ROC curve.** ROC, receiver operating characteristic; AUC, area under the curve.

The results of the ROC curve analysis of the PRL threshold level for predicting PAs in PCOS patients were as follows: the area under the ROC curve was 0.853 (95% confidence interval, 0.733-0.934; *P* < 0.001), and the sensitivity and specificity were 76.9% and 86.1%, respectively. The final cutoff value was 52.9 ng/mL (Fig. 2).

## Discussion

PAs associated with HPRL and PCOS were first documented in 1979. The authors of that report suggested that there might be some association between the 2 different disease entities 14. Since then, research on the correlation between HPRL/PA and PCOS has continued 4-8, 11-18.

Several studies provide a theoretical background for the increasing PRL level in PCOS. Mahboobifard et al. asserted that the pathway underlying PRL elevation in PCOS might be attributed to a decline in central dopaminergic tone associated with PCOS, which leads to an increase in levels of PRL. They tried to find out the upper reference level of PRL in PCOS patients and suggested adopting a value of about 1.5 times higher than normal for those <35 years of age 18. Luciano et al. postulated that hyperprolactinemia observed in a significant number of PCOS patients may reflect a greater deficiency of hypothalamic dopamine in these individuals 6. Işik et al. suggested that HPRL in PCOS is most likely related to a pathologic-endocrinologic milieu 4. However, other authors have argued that the association between HPRL and PCOS may be coincidental rather than a pathogenically related phenomenon 7, 15.

Some authors have suggested the possibility of a pathophysiological correlation between PA and PCOS. Kuroda et al. reported the case of a polycystic ovary being resolved following surgery to treat PAs in a study of patients with PCOS 11. Mashiro et al. reported 2 cases in which cystic pituitary adenoma and PCOS were confirmed to exist together, suggesting the possibility that continuous estrogenic stimulation of pituitary prolactin-secreting cells may be pathophysiology related to the co-existence of both diseases 12.

Delcour et al. reported the prevalence of hyperprolactinemia in PCOS patients according to the publication year of previous studies 15. The mean prevalence decreased from 28% to 19% with the publication of the Rotterdam criteria 3. In this study, it was 11.6% (76/657).

We focused on detecting PAs within hyperprolactinemic PCOS and identifying treatment strategies for these patients rather than the correlation between HPRL/PA and PCOS. Therefore, we tried to establish a cutoff value for the serum PRL level that could predict the presence of a PA. Kyristi et al. suggested a PRL cutoff value of 85.2 ng/mL for screening prolactinoma in PCOS patients according to the results of their study 8. In the present study, we identified a cutoff value of 53 ng/mL. Due to us evaluating the cutoff value among some cases of NFPA among 56 patients who underwent MRI, we believe a lower value than usual was derived from this analysis.

Macroprolactinemia is defined by the predominance of serum macroprolactin together with a non-pathologic monomeric PRL concentration 19. In previous studies 15, 17, authors suggested polyethylene glycol precipitation as a method to screen for macroprolactinemia. As described in our Methods section, the reagent used in our institution hardly detects macroprolactin, so the possibility of macroprolactinemia was excluded in our study.

Prolactinoma is the most common cause of HPRL, which accounts for 40% of all pituitary tumors 9. A PRL level elevation of > 250 ng/mL usually indicates the presence of a prolactinoma 19. It is difficult, however, to distinguish prolactinoma from NFPA solely by serum PRL level, especially in the case of a mildly to moderately elevated PRL level. Karavitaki et al. reported a borderline PRL level of 94 ng/mL between NFPA with HPRL and prolactinoma in their large series 17.

We believe that, when a PA is confirmed in a PCOS patient, a neurosurgeon should be involved, whether the PA is an NFPA or prolactinoma. Although dopamine-receptor agonists remain the first line of treatment for prolactinoma, unlike in the past, as technical developments have taken place, surgical intervention is now being re-evaluated as a treatment modality for prolactinoma 20. Some authors to date have reported medical treatments for NFPA 21-23.

It is not easy to treat a patient with both PA and PCOS. To provide the best outcome to patients, the hormone effects between the two separate endocrine organs, which are closely connected, should be considered, and each organ's treatment should be harmonized. In this study, the patients in group B (hyperprolactinemic PCOS with PAs) were all premenopausal. We accomplished normalization of the prolactin level and recovery of menstruation in 92.3% and 76.9% of patients, respectively. We have achieved these good outcomes through a multidisciplinary approach and care.

There are several limitations to this study. First, because this was a retrospective cohort study, there was no control group, and treatment strategies were heterogeneous. Therefore, we cannot suggest a specific guideline for the treatment of these patients. Second, since we aimed to identify PAs within hyperprolactinemic patients according to PRL level only, it was possible to overlook other factors affecting the results. A larger-scale and more elaborately designed study is needed to provide accurate diagnosis and treatment guidelines for hyperprolactinemic PCOS patients who may have PAs.

## Conclusion

In conclusion, PCOS patients with a PRL level of ≥ 52.9 ng/mL may need to consider undergoing sella MRI for PA detection. To help achieve a favorable clinical course for these patients, systematic diagnosis, treatment, and follow-up plan should be established. Therefore, a multidisciplinary approach is essential. This cutoff value should be confirmed through future studies. If a PCOS patient with HPRL is referred to a neurosurgeon, PA should be included in differential diagnosis for hyperprolactinemia.

## Ethics Committee Approval

The Institutional Review Board (IRB) of St. Vincent's Hospital approved this study design (IRB no. VC21RASI0183).

## Figures and Tables

**Table 1:** Clinicopathological characteristics of PCOS patients with HPRL (n = 56).

	HPRL without PA (n = 43)	HPRL with PA (n = 13)	*p* value
Age (years) Mean ± SD	27.73 ± 7.03	25.54 ± 6.32	0.540
BMI (kg/m^2^) Mean ± SD	23.14 ± 4.37	22.16 ± 2.50	0.460
PRL (ng/mL) Mean ± SD	39.89 ± 41.64	108.59 ± 60.70	< 0.001
Clinical HA (%)	25 (58.1)	8 (61.5)	0.085
Menstrual irregularity (%)	41 (95.3)	13 (100)	0.644
Polycystic ovary morphology (%)	40 (93.0)	13 (100)	0.293

PCOS, polycystic ovary syndrome; HPRL, hyperprolactinemia; PA, pituitary adenoma; SD, standard deviation; BMI, body mass index; PRL, prolactin; HA, hyperandrogenism.

## References

[1] Azziz R, Woods KS, Reyna R, Key TJ, Knochenhauer ES, Yildiz BO (2004). The prevalence and features of the polycystic ovary syndrome in an unselected population. The Journal of clinical endocrinology and metabolism.

[2] Fauser BC, Tarlatzis BC, Rebar RW, Legro RS, Balen AH, Lobo R (2012). Consensus on women's health aspects of polycystic ovary syndrome (PCOS): the Amsterdam ESHRE/ASRM-Sponsored 3rd PCOS Consensus Workshop Group. Fertility and sterility.

[3] Revised 2003 consensus on diagnostic criteria and long-term health risks related to polycystic ovary syndrome Fertility and sterility. 2004; 81: 19-25.

[4] Işik AZ, Gülekli B, Zorlu CG, Ergin T, Gökmen O (1997). Endocrinological and clinical analysis of hyperprolactinemic patients with and without ultrasonically diagnosed polycystic ovarian changes. Gynecologic and obstetric investigation.

[5] Lunde O (1981). Hyperprolactinaemia in polycystic ovary syndrome. Annales chirurgiae et gynaecologiae.

[6] Luciano AA, Chapler FK, Sherman BM (1984). Hyperprolactinemia in polycystic ovary syndrome. Fertility and sterility.

[7] Minakami H, Abe N, Oka N, Kimura K, Tamura T, Tamada T (1988). Prolactin release in polycystic ovarian syndrome. Endocrinologia japonica.

[8] Kyritsi EM, Dimitriadis GK, Angelousi A, Mehta H, Shad A, Mytilinaiou M (2018). The value of prolactin in predicting prolactinomicronma in hyperprolactinaemic polycystic ovarian syndrome. Eur J Clin Invest.

[9] Melmed S, Casanueva FF, Hoffman AR, Kleinberg DL, Montori VM, Schlechte JA (2011). Diagnosis and treatment of hyperprolactinemia: an Endocrine Society clinical practice guideline. The Journal of clinical endocrinology and metabolism.

[10] Karavitaki N, Thanabalasingham G, Shore HC, Trifanescu R, Ansorge O, Meston N (2006). Do the limits of serum prolactin in disconnection hyperprolactinaemia need re-definition? A study of 226 patients with histologically verified non-functioning pituitary macroadenoma. Clin Endocrinol (Oxf).

[11] Kuroda S, Yonekawa Y, Kawano T (1991). Pituitary prolactinoma associated with polycystic ovary-case report. Neurologia medico-chirurgica.

[12] Kurisaka M, Tindall SC, Takei Y (1983). Cystic prolactinoma of the pituitary following surgery for polycystic ovaries. Neurologia medico-chirurgica.

[13] Yavasoglu I, Kucuk M, Coskun A, Guney E, Kadikoylu G, Bolaman Z (2009). Polycystic ovary syndrome and prolactinoma association. Intern Med.

[14] Futterweit W, Krieger DT (1979). Pituitary tumors associated with hyperprolactinemia and polycystic ovarian disease. Fertility and sterility.

[15] Delcour C, Robin G, Young J, Dewailly D (2019). PCOS and Hyperprolactinemia: what do we know in 2019?. Clin Med Insights Reprod Health.

[16] Shilin DE, Mel'nichenko GA (1992). [Difficulties in the diagnosis and treatment of infertility caused by the combination of microprolactinoma and polycystic ovaries]. Probl Endokrinol (Mosk).

[17] Davoudi Z, Araghi F, Vahedi M, Mokhtari N, Gheisari M (2021). Prolactin Level in Polycystic Ovary Syndrome (PCOS): An approach to the diagnosis and management. Acta Biomed.

[18] Mahboobifard F, Rahmati M, Amiri M, Azizi F, Ramezani Tehrani F (2022). To what extent does polycystic ovary syndrome influence the cut-off value of prolactin? Findings of a community-based study. Adv Med Sci.

[19] Kasum M, Oreskovic S, Zec I, Jezek D, Tomic V, Gall V (2012). Macroprolactinemia: new insights in hyperprolactinemia. Biochem Med (Zagreb).

[20] Zamanipoor Najafabadi AH, Zandbergen IM, de Vries F, Broersen LHA, van den Akker-van Marle ME, Pereira AM (2020). Surgery as a Viable Alternative First-Line Treatment for Prolactinoma Patients. A Systematic Review and Meta-Analysis. The Journal of clinical endocrinology and metabolism.

[21] Even-Zohar N, Greenman Y (2018). Management of NFAs: medical treatment. Pituitary.

[22] Greenman Y, Cooper O, Yaish I, Robenshtok E, Sagiv N, Jonas-Kimchi T (2016). Treatment of clinically nonfunctioning pituitary adenomas with dopamine agonists. Eur J Endocrinol.

[23] Colao A, Di Somma C, Pivonello R, Faggiano A, Lombardi G, Savastano S (2008). Medical therapy for clinically non-functioning pituitary adenomas. Endocr Relat Cancer.

